# Impact of Exclusive Breastfeeding and Extrauterine Growth Restriction on Post-Discharge Growth in Preterm Infants: A Longitudinal Cohort Study in a Kangaroo Mother Care Program

**DOI:** 10.3390/children12050588

**Published:** 2025-04-30

**Authors:** Sergio Agudelo-Pérez, Diana Marcela Díaz Quijano, Eduardo Acuña, Juan Pablo Valderrama, Ariana Rojas

**Affiliations:** 1 Department of Pediatrics, School of Medicine, Universidad de La Sabana, Campus del Puente del Común, Km. 7, Autopista Norte de Bogotá, Chía 117068, Cundinamarca, Colombia; juanvalu@unisabana.edu.co (J.P.V.); arianaroor@unisabana.edu.co (A.R.); 2 Department of Epidemiology, School of Medicine, Universidad de La Sabana, Campus del Puente del Común, Km. 7, Autopista Norte de Bogotá, Chía 117068, Cundinamarca, Colombia; diana.diaz1@unisabana.edu.co; 3 PAIDEIA Research Group, Department of Pediatrics, Hospital Regional de Zipaquirá—Hospital Universitario de La Samaritana, Cra. 8 #0-29, Bogotá 110311, Colombia; hrzpediatria.coord@hus.org.co

**Keywords:** infant, premature, breast feeding, post-discharge nutrition and growth, extrauterine growth restriction

## Abstract

Background/Objectives: Extrauterine growth restriction (EUGR) and exclusive breastfeeding (EBF) are critical factors influencing early post-discharge growth in preterm infants. Although EBF is recommended in Kangaroo Mother Care (KMC) programs, its association with early anthropometric recovery remains unclear. This study evaluated the association between EUGR at 40 weeks of corrected age and EBF at 40 weeks, 3 months, and 6 months with anthropometric growth and acute malnutrition in preterm infants during the first six months of corrected age. Methods: A retrospective longitudinal cohort study was conducted, including 117 preterm infants (≤34 weeks of gestation) enrolled in the KMC program. Changes in weight, length, and head circumference z-scores and the incidence of acute malnutrition were analyzed using generalized estimating equations (GEEs). EUGR was defined as weight <10th percentile at 40 weeks. Acute malnutrition was defined as a weight-for-length z-score ≤−2. Results: EUGR was observed in 23.9% of the infants. EBF prevalence was 53% at 40 weeks and 40% at three and six months, respectively. EBF at 40 weeks was associated with a reduced weight z-score (coefficient: −0.29; *p* = 0.030), EBF at 3 months increased the weight z-score (coefficient: 0.34; *p* = 0.014), and EBF at 6 months reduced the risk of acute malnutrition (coefficient: −1.02; *p* = 0.036). Infants with EUGR showed greater weight gain over time (coefficient: 0.37; *p* = 0.020) yet remained below their non-EUGR peers. Conclusions: EBF during the first six months post-discharge supports weight gain and reduces the risk of malnutrition. However, EBF at 40 weeks may not ensure the immediate recovery of weight. EUGR is a key determinant of early growth.

## 1. Introduction

Approximately 15 million infants are born prematurely each year, representing 11% of all live births worldwide. This burden is particularly significant in low- and middle-income countries where access to adequate neonatal care is often limited [1]. Among preterm neonates, those born before 34 weeks of gestation or weighing less than 1500 g are at a higher risk of severe morbidities that hinder optimal nutritional support, often resulting in suboptimal postnatal growth and nutritional deficiencies [2]. These deficits not only impact their health during hospitalization but also frequently persist after discharge, compromising neurodevelopmental and metabolic outcomes in the short and medium terms [3].

A major consequence of inadequate early nutrition is extrauterine growth restriction (EUGR), defined as the failure to achieve the expected growth trajectories by the time of term-corrected age. EUGR is highly prevalent among preterm infants, with reported rates ranging from 14.9% to 71.6% [4,5].

Evidence suggests that EUGR may extend beyond discharge, potentially affecting future anthropometric development and increasing the risk of adverse neurodevelopmental and metabolic outcomes [6,7]. However, its role as a reliable predictor of long-term outcome remains controversial [8]. Regardless, early identification and targeted nutritional strategies during follow-up are essential to prevent persistent growth failure and associated complications [9].

Exclusive breastfeeding (EBF) is widely recommended as the optimal post-discharge feeding strategy for preterm infants, particularly in Kangaroo Mother Care (KMC) programs because of its immunological and developmental benefits [10]. The World Health Organization recommends exclusive breastfeeding for the first six months of life, given its association with multiple short- and long-term health benefits, including a reduced risk of infections, enhanced neurodevelopment, and lower risk of metabolic and cardiovascular diseases [11]. These benefits are particularly relevant for preterm infants, who are more vulnerable to growth and developmental challenges.

Nonetheless, some studies have questioned whether EBF alone is sufficient to meet the elevated nutritional demands of these infants, suggesting the potential need for breast milk fortification or specialized formulas to achieve adequate growth [12,13].

Despite growing interest, few studies have concurrently examined the impact of both EUGR and EBF on early post-discharge growth. The interactions between these factors remain insufficiently understood, especially within the context of structured KMC programs.

The present study aimed to evaluate the relationship between EUGR at 40 weeks of corrected age and feeding practices, specifically exclusive breastfeeding at 40 weeks, 3 months, and 6 months, and anthropometric growth and the incidence of acute malnutrition in a cohort of preterm infants. We hypothesized that the absence of EUGR and sustained EBF would be associated with improved growth outcomes and lower malnutrition rates during the first six months of corrected age.

## 2. Materials and Methods

This retrospective longitudinal cohort study included preterm neonates enrolled in the Kangaroo Mother Care (KMC) program at the Hospital Universitario de La Samaritana—Functional Unit, serving the Sabana Centro region in Cundinamarca, Colombia. To minimize potential biases associated with the COVID-19 pandemic, only neonates followed up between January 2017 and January 2019 prior to the pandemic were included. This study was approved by the Institutional Ethics Committee (Protocol ID: PI 2023-5).

Eligible participants were neonates born at ≤34 weeks of gestation, with appropriate weight for gestational age (10th–90th percentile, per Fenton growth charts), and assessed at birth using the Ballard method. Exclusion criteria included congenital anomalies, intrauterine growth restriction (IUGR), contraindications to breastfeeding, need for specialized formulas, feeding disorders, or requirement of alternative feeding methods.

### 2.1. Definitions and Key Variables

Gestational Age and Birth Weight: Gestational age was categorized as extremely preterm (≤28 weeks), very preterm (>28 to ≤32 weeks), or moderately preterm (>32 to ≤34 weeks). Birth weight was categorized as extremely low (≤1000 g), very low (>1000–≤1500 g), or low (>1500–<2500 g).

Corrected Age was calculated using the following formula: corrected age (months) = chronological age (months) − [(40 − gestational age at birth in weeks)/4], following AAP recommendations.

Growth and EUGR: Weight gain velocity (g/kg/day) from birth to 40 weeks was calculated as follows: (weight at 40 weeks − birth weight)/birth weight/days of life. Extrauterine growth restriction (EUGR) was defined as weight < 10th percentile at 40 weeks of corrected age, based on the Fenton growth calculator available online.

Follow-Up Schedule: Infants were monitored daily until weight gain ≥ 15 g/kg/day (<37 weeks) or 8–11 g/kg/day (until 40 weeks). After 40 weeks and weighing 2500 g, monthly visits were scheduled for the clinically stable neonates.

Anthropometry and Nutritional Status: Weight, length, and head circumference z-scores were assessed at 40 weeks, 3 months, and 6 months of corrected age using WHO Anthro software. Acute malnutrition was defined as a weight-for-length z-score ≤ −2.

Feeding Categories: At each visit, feeding practices were assessed via a 7-day recall by KMC pediatricians and classified as exclusive breastfeeding (EBF), mixed feeding, formula-only, or diversified feeding. Data were collected at corrected ages of forty weeks, three months, and six months.

Covariates: additional variables included sociodemographic data, maternal comorbidities, neonatal morbidities, ventilatory support type, and parenteral nutrition use.

Neonatal morbidity was defined as the presence of any of the following severe complications at the time of NICU discharge, based on criteria established in prior neonatal outcome studies: Severe intraventricular hemorrhage (IVH): grade III or IV intraventricular hemorrhage. Bronchopulmonary dysplasia (BPD): oxygen requirement at 36 weeks corrected gestational age. Necrotizing enterocolitis (NEC): Bell stage ≥ II. Retinopathy of prematurity (ROP): Stage ≥ 3 or requiring treatment.

Diagnoses were made according to standard clinical definitions and were confirmed through a review of medical records. Sepsis and surgical complications were recorded but were not included in the composite outcome of severe neonatal morbidity.

Fenton z-scores and percentiles were calculated using the validated online tool (https://ucalgary.ca/resource/preterm-growth-chart/calculators-apps, accessed on 15 November 2024), and the WHO z-scores were calculated using WHO Anthro software, version 3.2.2, available at the official WHO website (https://www.who.int/tools/child-growth-standards/software, accessed on 15 November 2024).

### 2.2. Statistical Analysis

Continuous variables were described using means and standard deviations (SD) or medians and interquartile ranges (IQR), according to the distribution assessed using the Kolmogorov–Smirnov test. Categorical variables were presented as absolute and relative frequencies.

Comparisons between neonates with and without extrauterine growth restriction (EUGR) were performed using Student’s *t*-test or Mann–Whitney U test for continuous variables and Chi-square or Fisher’s exact test for categorical variables, as appropriate.

Longitudinal changes in anthropometric z-scores (weight, length, and head circumference) from 40 weeks to 3 months and from 3 to 6 months of corrected age were analyzed using generalized estimating equations (GEEs), which accounted for within-subject correlation. An unstructured working correlation matrix was selected based on the lowest quasi-likelihood under the independence model criterion (QIC). Model specifications were assessed using residual analysis and QIC-based model selection to ensure the robustness of the fitted GEE models.

Additionally, GEE models were used to evaluate the association between feeding practices and the presence of acute malnutrition (defined as weight-for-length z-score ≤ −2) at 3 and 6 months of corrected age.

The main explanatory variables were EUGR at 40 weeks and exclusive breastfeeding (EBF) at 40 weeks, 3 months, and 6 months. These variables reflect time-specific exposure points and are not included simultaneously in a way that would result in multicollinearity, as they represent repeated measures over time.

Although birth weight < 1000 g and gestational age < 28 weeks were explored as additional covariates, they did not improve model fit according to the QIC and were therefore excluded from the final models for reasons of parsimony and model performance. This approach aligns with the criteria outlined previously.

The interaction terms between EUGR and EBF were explored; however, they showed no significant associations and were excluded from the final models. Covariates with *p* < 0.20 in univariate analysis were considered for multivariable models, with final selection based on QIC minimization.

Importantly, only infants without contraindications to breastfeeding or the need for specialized formulas were included in this study. However, not all infants maintained exclusive breastfeeding during follow-up; therefore, the EBF variables were treated as observed outcomes at each follow-up point and not as inclusion criteria.

All analyses were performed using STATA version 16. Statistical significance was defined as a two-sided *p*-value < 0.05.

## 3. Results

**Figure 1:**
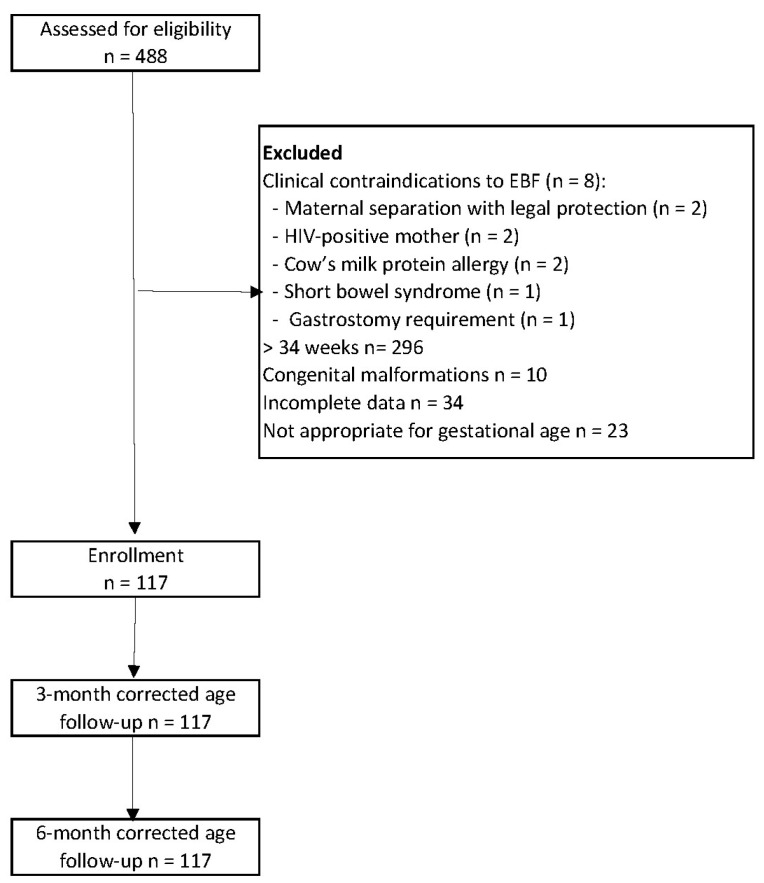
Flow chart of study.

In total, 117 preterm neonates were included in this study (Figure 1). The incidence of extrauterine growth restriction (EUGR) at 40 weeks of corrected age was 23.9% (n = 28). The prevalence of exclusive breastfeeding (EBF) was 53% (n = 62) at 40 weeks, decreasing to 40% (n = 47) at both 3 and 6 months of corrected age. The incidence of acute malnutrition was 17.9% (n = 21) at three months and 6.8% (n = 8) at six months.

Comparisons between neonates with and without EUGR showed significant differences in gestational age (*p* < 0.001), birth weight (*p* < 0.001), presence of neonatal morbidity (*p* = 0.01), and weight gain velocity during hospitalization (*p* < 0.001). Most infants with EUGR had very low birth weights (1000–1500 g), whereas the majority of non-EUGR infants had low birth weights (>1500 g), as shown in Table 1.

**Figure 2:**
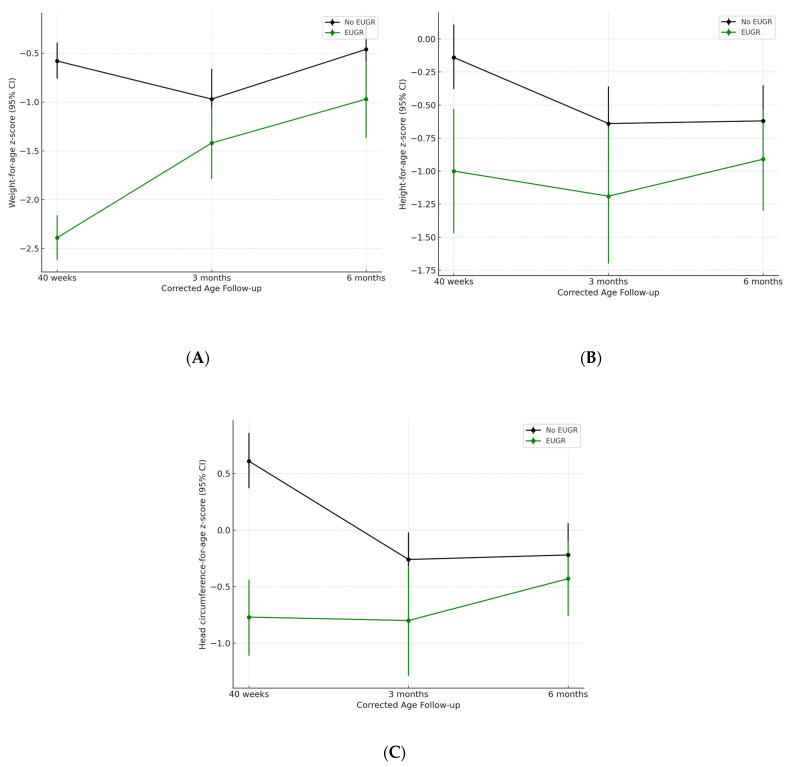
Evolution of z-score for weight, length, and head circumference during the first six months of corrected age. (**A**). Weight z-score. (**B**). Length z-score. (**C**). Head circumference z-score. EUGR: extrauterine growth restriction.

Anthropometric trajectories differed notably between the groups. Neonates with EUGR had consistently lower z-scores for weight, length, and head circumference than those without EUGR at all time points. Although the weight z-scores improved over time in the EUGR group, they remained below those of their non-EUGR counterparts at six months of corrected age (Figure 2A–C).

Generalized estimating equations (GEEs) showed that EUGR was significantly associated with a greater increase in weight-for-age z-scores from 40 weeks to 6 months. Exclusive breastfeeding showed time-dependent effects; at 40 weeks, it was associated with a slight decrease in weight z-score, whereas at 3 months, it was positively associated with weight gain (Table 2).

Male sex was the only variable significantly associated with an increase in the length-for-age z-score during the follow-up (coefficient, 0.52; 95% CI: 0.24–0.80; *p* < 0.001). Neither EUGR nor EBF showed a significant association with linear growth.

In terms of head circumference, both EUGR and male sex were associated with greater increases in z-scores during the follow-up. EUGR was associated with an increase in 0.51 z-score units (95% CI: 0.24–0.79; *p* < 0.001), and male sex was associated with an increase of 0.50 units (95% CI: 0.23–0.78; *p* < 0.001) (Table 3).

Finally, EBF at six months was significantly associated with a reduced risk of acute malnutrition (coefficient: −1.02; 95% CI: −1.97, −0.07; *p* = 0.036) (Table 4).

## 4. Discussion

This study assessed anthropometric growth trajectories and the occurrence of acute malnutrition in preterm infants (≤34 weeks of gestation) without intrauterine growth restriction during the first six months of corrected age. Our findings revealed that infants with extrauterine growth restriction (EUGR) at 40 weeks showed greater improvement in weight z-scores over time, suggesting compensatory catch-up growth. Exclusive breastfeeding (EBF) at three months was positively associated with weight gain, whereas EBF at six months was associated with a reduced risk of acute malnutrition. In contrast, neither EUGR nor EBF influenced changes in length-for-age z-scores. Interestingly, both EUGR and male sex were associated with greater increases in head circumference z-scores, indicating potentially divergent growth patterns of different anthropometric dimensions.

These results underscore the role of EBF in promoting healthy weight recovery and preventing malnutrition, particularly in the Kangaroo Mother Care (KMC) programs. The observed high prevalence of EBF in this cohort exceeded the national rates for term infants [14], highlighting the potential of structured follow-up and breastfeeding support strategies. The high incidence of EUGR emphasizes the need for systematic growth monitoring and individualized nutritional interventions after discharge.

Our findings are consistent with those of Lan et al. [15], who reported greater weight gain in infants with EUGR than in their peers. However, Peila et al. [16] and Kim et al. [17] observed long-term weight growth limitations among EUGR infants in contrast to our short-term improvements. Lan’s study also raised concerns about the risk of being overweight owing to accelerated catch-up growth, a phenomenon that may pose long-term metabolic risks.

These observations reinforce the importance of achieving harmonious growth rather than rapid weight gain alone, as emphasized in KMC guidelines [18]. Although catch-up growth can mitigate short-term undernutrition, excessive velocity may predispose individuals to adverse metabolic outcomes later in life [15,19]. Therefore, careful monitoring is essential to ensure that growth is steady, proportionate, and sustained across weight, length, and head circumference dimensions.

Nutrition remains the cornerstone of post-discharge care in preterm infants. Our data support the EBF value during the first month and beyond, showing a positive association between weight gain and protection against acute malnutrition. However, the findings in the literature remain mixed. Zhang et al. [9] demonstrated superior weight outcomes after breast milk fortification and formula supplementation, whereas Vesel et al. [20] found that EBF had no significant effect on reducing underweight, wasting, or stunting. Zhang et al. associated EBF with better neurodevelopmental outcomes, reinforcing its value beyond physical growth. Wang et al. [21] found that preterm infants receiving KMC had higher rates of EBF and improved physical growth, supporting the integrative effect of breastfeeding and KMC environment.

Collectively, the available evidence, including our findings, supports prioritizing early and adequate nutrition in KMC programs, and balancing the benefits of EBF with the potential need for fortification in selected cases. Optimal growth in this population should not be defined solely by weight gain but also by stability and proportion across all anthropometric parameters, minimizing long-term cardiometabolic risks.

A final consideration involves the variability in the operational definitions of EUGR. While our study employed a cross-sectional definition (weight <10th percentile at 40 weeks), other studies used longitudinal criteria based on changes in z-scores from birth [22,23]. This heterogeneity complicates comparisons across studies and highlights the need to standardize diagnostic criteria to improve consistency in research and clinical care.

This study has several limitations. The retrospective design may have introduced biases related to data availability and measurement accuracy. Although standardized criteria from the KMC program were applied, the influence of residual confounding factors cannot be entirely excluded. Moreover, the six-month follow-up period limits the ability to assess long-term outcomes related to neurodevelopment and metabolic health. Additionally, while z-scores and changes in z-scores were used as standardized measures of growth, these metrics may not fully capture the dynamic nature of growth, such as fluctuations in growth velocity or changes in body composition over time.

Nonetheless, the strengths of this study include the use of a well-defined cohort with a comprehensive anthropometric follow-up and rigorous statistical modeling. Furthermore, the detailed evaluation of EBF across multiple time points provides valuable insights into its protective role in high-risk neonatal populations.

## 5. Conclusions

In conclusion, this study highlights the beneficial role of exclusive breastfeeding (EBF) in promoting weight gain and reducing the risk of acute malnutrition during the first six months of corrected age in preterm infants. These findings support the continued promotion of EBF as a core strategy for post-discharge nutritional support in Kangaroo Mother Care programs. Moreover, the observed variability in the definitions of extrauterine growth restriction (EUGR) and their influence on clinical outcomes underscores the urgent need for standardized diagnostic criteria. Establishing a unified framework would facilitate more consistent monitoring, risk stratification, and long-term follow-up in this vulnerable population.

## Figures and Tables

**Table 1:** Sociodemographic and clinical characteristics of mothers and newborns according to the presence of extrauterine growth restriction.

Characteristic	Total (n = 117)	EUGR		*p*-Value
		Yes (n = 28)	No (n = 89)	
*Maternal characteristic*				
Age (years), median—IQR	25 (8)	25 (9)	25 (8)	0.91 *
Educational level, n (%)				
Primary	48 (41.0)	11 (39.3)	37 (41.6)	0.06
Secondary	50 (42.7)	16 (57.1)	34 (38.2)	
Technical/professional	19 (16.3)	1 (3.6)	18 (20.2)	
Occupation, n (%)				
Homemaker/Student	89 (76.1)	22 (78.6)	67 (75.3)	0.73
Technical	20 (17.1)	5 (17.9)	15 (16.9)	
Independent/professional	8 (6.8)	1 (3.6)	7 (7.9)	
Marital status, n (%)				
Cohabiting/Married	106 (90.6)	78 (87.6)	28 (100)	0.06
Divorced/not living together	11 (9.4)	11 (12.4)	0	
Maternal morbidity, n (%)				
No	77 (65.8)	18 (64.3)	59 (66.3)	0.84
Yes	40 (34.2)	10 (35.7)	30 (33.7)	
Prenatal care, n (%)				
None	6 (5.1)	2 (4.5)	4 (7.1)	0.13
≤3	29 (24.8)	3 (29.2)	26 (10.7)	
≥4	82 (70.1)	23 (66.3)	59 (82.1)	
*Newborn characteristic*				
Sex, n (%)				
Female	48 (41.1)	8 (44.9)	40 (28.6)	0.12
Male	69 (58.9)	20 (55.1)	49 (71.4)	
Birth methods, n (%)				
Vaginal	51 (43.6)	9 (47.2)	42 (32.1)	0.16
Cesarean	66 (56.4)	19 (52.8)	47 (67.9)	
Neonatal resuscitation at birth, n (%)				
No	84 (71.8)	19 (67.9)	65 (73.0)	0.59
Yes	33 (28.2)	9 (32.1)	24 (27.0)	
Head circumference at birth (CMS), median—IQR	30.2 (2.5)	29.9 (3)	30.9 (2)	0.009 *
Gestational age, n (%)				
Moderate preterm (>32–≤34 weeks)	74 (63.2)	9 (32,1)	65 (73.0)	<0.001
Very preterm (>28–≤32 weeks)	36 (30.8)	14 (50.0)	22 (24.7)	
Extreme preterm (≤28 weeks)	7 (6.0)	5 (17.9)	2 (2.3)	
Birth weight, n (%)				
Low birth weight (>1500–<2500 g)	93 (79.4)	12 (42.9)	81 (91.0)	<0.001
Very low birth weight (>1000–≤1500 g)	21 (18.0)	14 (50.0)	7 (7.9)	
Extremely low birth weight (≤1000 g)	3 (2.6)	2 (7.1)	1 (1.1)	
Neonatal morbidity, n (%)				
No	62 (53.0)	9 (32.1)	53 (59.6)	0.01
Yes	55 (47.0)	19 (67.9)	36 (40.4)	
Type of respiratory support during hospitalization, n (%)				
None	65 (55.6)	15 (53.6)	50 (56.3)	0.51
Invasive ventilation	21 (17.9)	5 (17.9)	16 (17.8)	
No invasive	5 (4.3)	0 (0)	5 (5.7)	
Nasal cannula	26 (22.2)	8 (28.5)	18 (20.2)	
Parenteral nutrition				
No	96 (82.1)	22 (78.6)	74 (83.1)	0.58
Yes	21 (17.9)	6 (21.4)	15 (16.9)	
Weight gain rate (g/kg/day), median—IQR	15.4 (10.5)	8.1 (4.0)	17.8 (10)	<0.001 *

* Mann–Whitney U test. EUGR: extrauterine growth restriction; IQR: interquartile range.

**Table 2:** Association between EUGR, exclusive breastfeeding, and weight-for-age z-score changes from 40 weeks to 6 months corrected age (generalized estimating equation model).

Change in Weight Z-Score	Unadjusted Coefficient	95% CI		*p*-Value	Adjusted Coefficient	95% CI		*p*-Value
Extrauterine Growth Restriction	0.64	0.38	0.91	<0.001	0.37	0.06	0.68	0.02
Exclusive Breastfeeding at 40 Weeks Corrected Age	−0.21	−0.46	0.03	0.09	−0.29	−0.55	−0.03	0.03
Exclusive Breastfeeding at 3 Months Corrected Age	0.14	−0.11	0.41	0.28	0.34	0.07	0.61	0.01
Neonatal Morbidity at Discharge from NICU	0.33	0.08	0.58	0.008	0.26	0.02	0.50	0.03
Weight Gain Rate (g/kg/day)	−0.02	−0.04	−0.01	<0.001	−0.02	−0.03	0.00	0.03
Constant					0.33	−0.05	0.71	0.09

**Table 3:** Association between EUGR, sex, and head circumference z-score changes from 40 weeks to 6 months corrected age (generalized estimating equation model).

Change in Head Circumference Z-Score	Unadjusted Coefficient	95% CI		*p*-Value	Adjusted Coefficient	95% CI		*p*-Value
Extrauterine Growth Restriction	0.59	0.31	0.87	<0.001	0.51	0.24	0.79	<0.001
Sex	0.56	0.27	0.85	<0.001	0.50	0.23	0.78	<0.001
Constant					−0.70	−0.93	−0.46	<0.001

CI: confidence interval.

**Table 4:** Generalized estimating equation model for acute malnutrition in the first six months of corrected age.

Acute Malnutrition	Coefficient	Standard Error	95% CI		*p*-Value
Exclusive Breastfeeding at 6 Months of Corrected Age	−1.02	0.49	−1.97	−0.07	0.03
Constant	−1.64	0.26	−2.16	−1.13	<0.001
CI: confidence interval					

## Data Availability

The data presented in this study are not publicly available because of ethical and privacy restrictions. However, de-identified data may be made available upon reasonable request to the corresponding author, subject to approval by the Institutional Ethics Committee, and in compliance with data protection regulations.

## Abbreviations

The following abbreviations are used in this manuscript:

EUGR	Extrauterine growth restriction
EBF	Exclusive breastfeeding
KMCK	Kangaroo Mother Care
NICU	Neonatal intensive care unit
GA	Gestational age
SD	Standard deviation
IQR	Interquartile range
GEE	Generalized estimating equation
QIC	Quasi-likelihood under the independence Model criterion
WHO	World Health Organization
AAP	American Academy of Pediatrics
CI	Confidence interval
HC	Head circumference
WLZ	Weight-for-length z-score
LBW	Low birth weight
VLBW	Very low birth weight
ELBW	Extremely low birth weight
IUGR	Intrauterine growth restriction
